# The impact of gender of the examiner on orofacial pain perception and pain reporting among healthy volunteers

**DOI:** 10.1007/s00784-021-04286-9

**Published:** 2021-12-13

**Authors:** A. Lövgren, B. Häggman-Henrikson, A. Fjellman-Wiklund, A. Begic, H. Landgren, V. Lundén, P. Svensson, C. Österlund

**Affiliations:** 1 Faculty of Medicine, Department of Odontology, Clinical Oral Physiology, Umeå University, 901 87 Umeå, Sweden; 2 Department of Orofacial Pain and Jaw Function, Faculty of Odontology, Malmö University, Malmö, Sweden; 3 Department of Community Medicine and Rehabilitation, Physiotherapy, Umeå University, Umeå, Sweden; 4 Section for Orofacial Pain and Jaw Function, Department of Dentistry and Oral Health, Aarhus University, Aarhus, Denmark

**Keywords:** Orofacial pain, Experimental pain, Pressure pain, Pain perception, Pain reporting, Gender roles

## Abstract

**Objectives:**

Pain on palpation of jaw muscles is a commonly used diagnostic criterion when examining patients with orofacial pain. It is not known, however, if pain reports are affected by the gender of the examiner. Our aim was to investigate if pressure pain threshold (PPT), pressure pain tolerance (PTol), and pain intensity assessed over the masseter muscles in healthy individuals are affected by the gender of the examiner.

**Materials and methods:**

Healthy, pain-free individuals were recruited on a voluntary basis. PPT and PTol were assessed using pressure algometry. At the PTol level, participants also rated pain intensity on a 0–10 numeric rating scale. Assessments of PPT and PTol were conducted with six repeated measurements performed twice, separately by one female and one male examiner, on each participant.

**Results:**

In total, 84 participants (43 women; median age 24, IQR 6) were included. With a female examiner, women reported higher pain intensity than men (Mann Whitney *U*, *p* = 0.005). In the multivariable analysis, significantly higher PTol was predicted by male examiner. Also, a higher ratio between PTol and reported pain intensity was predicted by male examiner.

**Conclusions:**

The gender of the examiner influences pain reporting and perception in an experimental setting. This effect on pain perception related to gender of the examiner is probably related to normative gender behaviors rather than to biological alterations within the examined individual.

**Clinical relevance:**

In clinical and experimental settings, gender of the examiner may affect not only pain perception but also pain reporting, with potential implications for diagnostics in patients with pain.

## Background

### Pain perception

In clinical practice, palpation of anatomical structures is a basis for diagnostics of pain complaints [1], with pain reported during palpation interpreted as a positive test outcome. Pain perception describes the response to unpleasant sensation due to actual or potential tissue damage, or in terms of such damage [2]. The individual pain perception consists of three basic components: the nociceptive component, i.e., pain localization and intensity, the emotional response due to unpleasantness, and the cognitive response guided by previous experiences and expectations [3]. Thus, the intrinsic pain modulating system comprises multiple regions of the central nervous system influenced by both physical and psychological factors. Among the psychological factors related to pain perception are the emotional state such as anxiety and depression, the attention given to the injury, and the experience of previous injuries [4]. Therefore, the response to a stimulus may differ for a standardized nociceptive stimulus. One such example is gender differences in pain perception identified in both experimental and clinical settings [5].

### Sex, gender, and pain

Differences between women and men in relation to pain seem to be related to both sex and gender. The concept of sex refers to the biological aspects of being a woman or a man whereas gender is regarded as a uniquely human concept based on a person’s self-representation as woman or man, and rooted in biology as well as in the individual’s environment and experiences [5, 6].

Most pain conditions, including orofacial pain [7], have a higher prevalence among women than men [8]. In addition, women report more intense pain, a longer duration of pain, and more frequent pain [9]. The reasons for these gender differences are not known, but could be related to physical and psychological differences, or combinations of both. The importance of normative gender behaviors in relation to pain is debated [10]. Gender role expectations are described as the learned roles into feminine and masculine behaviors. These socialized behaviors are suggested to have an impact on pain reporting [5], often affirming that men should be tough and stoic, i.e., a perceived sign of being impervious to pain. In this context, also the gender of the examiner might influence how experimentally induced pain is perceived and reported.

Orofacial pain complaints are managed on a daily basis in clinical dental practice. With a prevalence of approximately 10% among adults [7, 11, 12], chronic orofacial pain is most commonly related to temporomandibular disorders (TMD) [13]. Since diagnostics of TMD pain is made partly by palpation of the jaw muscles, this could be used as a model to evaluate the possible impact of gender of the examiner on pain perception and pain reporting. Even though palpation is used in the clinical situation, pressure algometry is often used in experimental settings as a standardized, quantitative evaluation of mechanical sensitivity of a tissue including pain perception and reporting [14]. To our knowledge, the possible impact of gender of the examiner on pressure pain threshold (PPT) and pressure pain tolerance (PTol) assessed over the masseter muscles using pressure algometry is unknown.

Therefore, our first aim was to investigate the representativeness of reported PPT, PTol, and pain intensity assessed over the masseter muscles in healthy individuals in an experimental setting. Our second aim was to investigate if gender of the examiner impacted the reported pressure pain. The hypothesis was that gender of the examiner influences PTol but not PPT.

## Materials and methods

### Participants

Participants were recruited among students at Umeå University, Sweden, by advertising in public areas on the campus and on Facebook. In addition, information regarding the study was provided during visits to lectures given to dental and medical students. Data was collected from September 2018 to January 2020.

The inclusion criteria were healthy individuals aged 18–40 years with no pain in the jaw system, head, neck, shoulders, or back regions. The exclusion criteria were a positive response to any of the screening questions (3Q/TMD) on frequent pain in the orofacial area or pain on jaw function [15], the presence of orofacial pain diagnosis according to the established Diagnostic Criteria for TMD—DC/TMD (arthralgia, myalgia, headache contributed to TMD) [1], local or generalized pain conditions, or history of trauma to the head, face, jaws, or neck that had caused persistent pain. Also excluded were individuals with neurological, inflammatory/rheumatic, autoimmune disorders, fibromyalgia, or cardiovascular disease, as well as those regularly using analgesics.

Eligibility of potential participants was assessed with a screening questionnaire. The clinical examination according to the DC/TMD was performed directly preceding the data collection by one single trained male examiner to ensure that the participants did not meet a DC/TMD pain diagnosis.

**Fig. 1:**
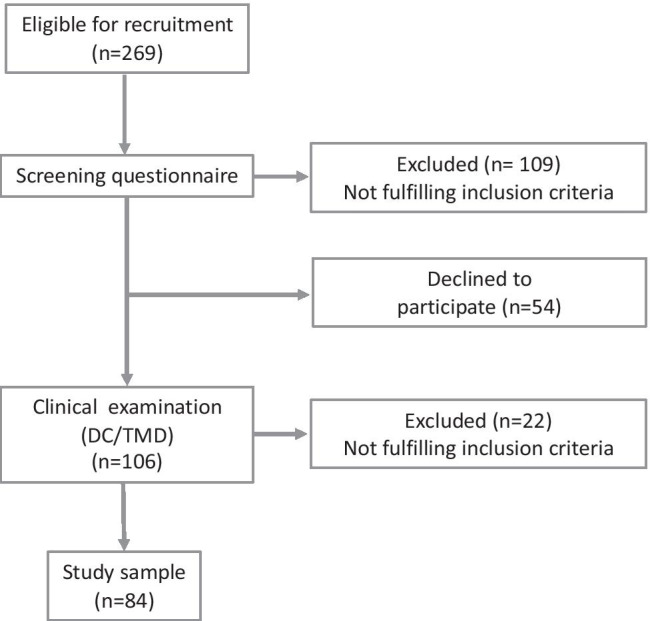
Flow chart of recruitment process of the study sample (*n* = 84)

A total of 269 participants were eligible, of which 109 were excluded at the screening stage, due to meeting at least one of the exclusion criteria. An additional 54 participants could not be reached or chose to withdraw. This resulted in 84 participants included in the study sample (Fig. 1).

The study was approved by the Regional Ethical Board in Umeå, Sweden (Dnr 2013/328-31 M and Dnr 2018/259-32 M). All participants signed a written informed consent prior to the data collection.

### Outcome variables

PPT was defined as the lowest pressure in kilopascal (kPa) that a participant reports as painful [16, 17]. PTol was defined as the maximum amount of pressure (kPa) that a participant was willing or able to accept [16]. An upper limit for the measurement of PTol was set at 700 kPa [18]. All measurements were performed bilaterally over the central part of the masseter superficial muscle belly. A mark made on the skin with a water-soluble felt pen ensured intra- and inter-examiner consistency of the location of the application of the algometer probe.

Pain intensity was reported at the PTol level by the participant pointing to a numeric rating scale (NRS) ranging from 0 to 10, where 0 represents no pain and 10 represents the worst possible pain [19].

### Experimental design and procedures

The participants were required to abstain from analgesics [14] and alcohol for 24 h prior to the examination. Each participant was greeted and led to the examination room by the examiner performing the clinical screening examination. The participant received verbal information about the procedures of the examination and the subsequent data collection. All communication followed a standardized script. Participants did not receive any information about the aim before, during, or after the test session. The clinical examination followed the DC/TMD protocol. The experimental procedure followed a strict study protocol that was developed based on available guidelines [14]. The DC/TMD examination and data collection were performed in the same undisturbed and quiet room, with only the participant and the examiner present during the procedure. The participant was seated in an upright position in a chair with a backrest, headrest, and armrests.

For each participant, assessments of PPT and PTol were performed twice, separately by one female and one male examiner. Each examiner performed six measurements, three on the masseter muscle of each side, always starting with the right masseter muscle and then alternating between the sides. There was a 1-min resting period between each measurement [14], and a 5-min resting period before the second examiner started data collection. Five minutes was considered an appropriate time for the participant to rest and refocus. For each participant, the gender of first examiner was randomized just prior to the data collection procedure.

An electronic pressure algometer (Somedic AB, Sweden) with a 1 cm^2^ circular probe was used. The algometer was calibrated by each examiner before every first examination for each participant. On both sides, pressure was applied on the central part of the masseter superficial muscle belly at an increasing pressure rate of 30 kPa/s (9).

When applying pressure, the examiner placed one hand on the other side of the participant’s head to ensure stability, and the participant was instructed to relax their jaw for the duration of the measurement. Each measurement required the participant to press and hold a button at the first perception of pain to mark the PPT, and then release the button when the pain could no longer be tolerated (PTol). PPT and PTol values were displayed on the screen connected to a computer with the Somedic software. To avoid bias, the monitor displaying the collected data was positioned outside the visual field of the participant. For each measurement, the participant was tasked to rate the pain intensity on the NRS scale at the point of PTol.

### Statistical methods

Descriptive statistics were used to characterize the study sample. The normality of the data was assessed by descriptive statistics, visual inspection of histograms, and the Shapiro–Wilk test. Comparisons between groups were evaluated by using non-parametric statistics with Mann Whitney *U* and Wilcoxon Signed rank for repeated measures. Each participant’s first measurement was excluded [14]. From the five remaining measurements, the mean score for PPT, PTol, and NRS was calculated for each participant. All PTol values exceeding the limit were set to 700 kPa. To evaluate the relationship between pain tolerance and pain intensity ratings, the ratio between the mean value of PTol and the mean value of the reported pain intensity for each individual examination was calculated. The coefficient of variation (CV) was calculated to evaluate the intra-individual variability between repeated measures (*n* = 5). The correlation between PTol and pain intensity was evaluated with Spearman’s correlation. An unstructured, linear generalized estimating equation accounting for repeated measures was used to evaluate whether the gender of the examiner predicted the reporting of pain perception (PTol and ratio PTol/NRS, respectively). In a second step, the model was adjusted for the effect of gender of participant together with the multiplicative interaction term between gender of participant and gender of the examiner.

### Sample size calculation

After data collection of an initial sample of 36 participants, a sample size calculation was performed based on the available data and a standard deviation of 90 kPa at PTol. To detect a difference between groups of 60 kPa, representing a pressure lasting for 2 s and being comparable to diagnostics for myofascial orofacial pain based on palpation [1]. The total sample size was calculated at 72 participants, which was deemed satisfactory for the purpose of this study.

Statistical analyses were performed in SPSS and GraphPad Prism. A p-value < 0.05 was considered statistically significant.

## Results

In total, 84 participants (43 women; median age 24, IQR 6) were included. There was no significant difference in age between women and men (Mann Whitney *U* 706.0, *p* = 0.114). A female examiner performed the first examination in 70 out of the 168 examinations conducted in 84 individuals. In seven participants (ten measurements in total), no PTol measurements were available for analysis since pain tolerance levels were not reached before the 700 kPa limit.

Women reported significantly lower PPT with both male and female examiners (Mann Whitney *U*, *p* = 0.005 male and *p* < 0.001 female examiner) and PTol values (*p* = 0.006 male and *p* = 0.001 female examiner) compared to men (Table 1). The variability in PTol expressed as CV was significantly higher with a male examiner in women compared to men (Mann Whitney *U*, *p* = 0.007) (Table 2).

**Table 1:** Pressure pain threshold (PPT), pressure pain tolerance (PTol), pain intensity on a numerical rating scale (NRS), and ratio PTol/NRS together with inter quartile range (IQR) in women and men, respectively

		**PPT (kPa)**			**PTol (kPa)**			**Pain intensity (NRS)**			**Ratio PTol/NRS**		
		Female examiner	Male examiner	*p*-value^3^	Female examiner	Male examiner	*p*-value^3^	Female examiner	Male examiner	*p*-value^3^	Female examiner	Male examiner	*p*-value^3^
**Women**^1^	Median (IQR)	169.1 (80.6)	161.0 (83.5)	0.348	456.0 (192.6)	462.2 (193.4)	**0.007**	8.0 (1.4)	8.0 (1.6)	0.075	55.8 (27.3)	59.1 (30.6)	**0.006**
**Men**^2^	Median (IQR)	229.0 (111.4)	191.9 (101.9)	** < 0.001**	556.6 (121.0)	555.9 (163.9)	0.512	7.4 (1.8)	7.6 (1.4)	0.378	74.3 (40.6)	78.7 (28.2)	0.623
	*P*-value^4^	** < 0.001**	**0.005**		** < 0.001**	**0.006**		**0.004**	0.065		** < 0.001**	**0.001**	

^1^Women (PPT *n* = 43; PTol and NRS *n* = 39 male examiner; PTol and NRS *n* = 41 female examiner)

^2^Men (PPT *n* = 41; PTol and NRS *n* = 39)

^3^Wilcoxon matched pairs signed rank test

^4^Mann Whitney *U* test

**Table 2:** The coefficient of variation (CV) calculated for pressure pain threshold (PPT) and pressure pain tolerance (PTol), in women and men, respectively

	**CV PPT (%)**			**CV PTol (%)**		
	Female examiner	Male examiner	*p*-value^3^	Female examiner	Male examiner	*p*-value^3^
**Women**^1^	15	16	0.223	9	12	0.091
**Men**^2^	18	19	0.957	8	9	0.660
*P*-value^4^	0.068	0.423		0.100	**0.007**	

^1^Women (PPT *n* = 43; PTol *n* = 39 male examiner; PTol *n* = 41 female examiner)

^2^Men (PPT *n* = 41; PTol *n* = 39)

^3^Wilcoxon matched pairs signed rank test

^4^Mann Whitney *U* test

**Fig. 2:**
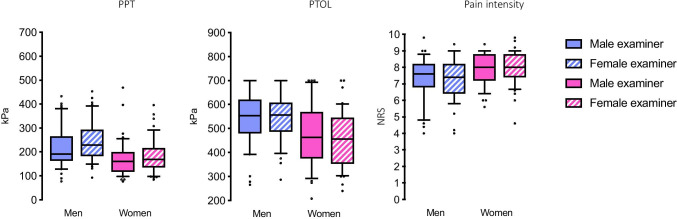
Pressure pain threshold (PPT), pressure pain tolerance (PTol), and pain intensity on the numerical rating scale (NRS) reported by men and women when assessed by male and female examiners, respectively. The box plots illustrate the medians, interquartile ranges and the 10th and 90th percentiles. Dots represent values outside the 10th and 90th percentiles

**Fig. 3:**
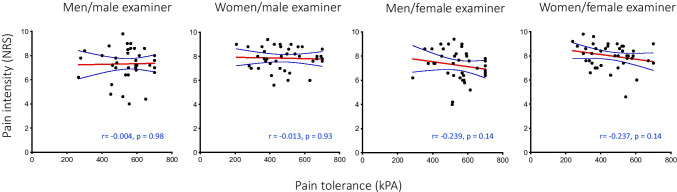
Scatterplot between pain tolerance and pain intensity on the numerical rating scale (NRS) reported by men and women when assessed by male and female examiners, respectively: (**a**) men/male examiner; (**b**) women/male examiner; (**c**) men/female examiner; **d**) women/female examiner. No significant correlations were found (Spearman’s rho)

Women reported higher pain intensity at PTol compared to men with a female examiner, (Mann Whitney *U*, *p* = 0.004) whereas there was no statistically significant difference with a male examiner (Mann Whitney *U*, *p* = 0.065) (Table 1, Fig. 2). Correlations are presented in Fig. 3.

The ratio between PTol and the reported pain intensity was significantly higher for men compared to women with both male and female examiners (Mann Whitney *U*, *p* = 0.001 male, and *p* < 0.001 female examiner, respectively).

In the multivariable analysis, significantly higher PTol, and a higher ratio between PTol and reported pain intensity were predicted by a male examiner (Table 3). No significant interaction between the gender of participant and the gender of the examiner was found.

**Table 3:** Pain reporting in the study sample predicted by the gender of examiner

Dependent variable	Independent variable	Univariable analysis		Multivariable analysis	
		$\beta$	*p* value	$\beta$	*p* value
Pain tolerance	Intercept	499.67	< 0.001	456.46	< 0.001
	Male examiner	16.85	0.02	24.63	0.002
	Male participant			87.82	< 0.001
	Gender interaction^1^			-16.04	0.27
Ratio pain tolerance and pain intensity	Intercept	68.27	< 0.001	58.61	< 0.001
	Male examiner	2.39	0.10	4.13	0.021
	Male participant			19.64	< 0.001
	Gender interaction^1^			-3.64	0.21

^1^Interaction gender examiner and gender participant

## Discussion

The main finding from this study in healthy individuals was that gender differences in pain perception and pain reporting are associated with the gender of the examiner. In this experimental setting, women tolerated significantly higher painful pressure stimuli and the variability between repeated measures of PTol was higher with a male examiner. In addition, the ratio between PTol and reported pain intensity was predicted by a male examiner. For pain intensity on its own, women reported higher pain intensity scores than men with a female, but not with a male examiner.

### Reported values in relation to previous studies

In line with previous findings, female participants in our study reported lower PPTs compared to males [5, 14]. Furthermore, reported mean PPT and PTol were in accordance with previously reported data for the masseter muscles in healthy women and men (PPT women 194.1 ± 62.7 kPa and men 248.2 ± 48.4 kPa, PTol women 353 ± 111 and men 572 ± 380), which lends further credibility to the accuracy of our measurements [14, 20–22]. Taken together, our findings on differences in pain perception among men and women together with representative values on PPT and PTol indicate that this experimental set-up is appropriate for future studies of pain reporting in relation to gender of the examiner.

### Pain reporting in relation to gender of the examiner

The largest impact of gender of the examiner, with a magnitude of potential clinical relevance, was found for pain perception in men with higher PPT with a female examiner. These findings indicate both a reluctance of reporting pain to the opposite gender as well as being more comfortable to report pain to the same gender. Moreover, the ratio between PTol and reported pain intensity was significantly higher in men compared to women indicating a greater difference in between pain tolerance and pain intensity. For pain intensity alone, women reported significantly higher pain intensity than men with a female examiner. All in all, these findings suggest a higher ability in men to tolerate pain, thereby reinforcing previous reports of men being more stoic and less willing to report pain [10], whereas women are socialized to verbalize discomfort [23].

Even after adjusting for the effect of participants’ gender on pain tolerance, a positive association remained between gender of the examiner and both pain tolerance and the ratio between pain tolerance and pain reporting. The negative but non-significant interaction term evaluated in the model could be a consequence of either no actual interaction or a too small sample to being able to detect a possible interaction. In this setting, and even though gender of the examiner still has a significant impact, gender of the participant still has the strongest predictive impact. Furthermore, the fact that men and women responded differently in relation to the gender of the examiner could also affect the outcome of the interaction term.

Individual variability in pain reporting in itself could be regarded a measure of the consistency of the somatosensory system [24] There is, however, no clear biological rationale for this variability being influenced by the gender of the examiner. Interestingly though, we did find a larger PTol variability with a male examiner. Taken together, this reinforces that pain reporting in general should be regarded complex and not only related to sensory-discriminative components of the nociceptive stimulus but also related to the surrounding context and to cognitive and behavioral aspects of pain processing. This in turn may have significant implications both in experimental studies and in clinical practice.

### Interpretation of results

Previous findings provide inconclusive evidence on whether the gender of the examiner affects the perception of experimentally induced pain [25]. To the best of our knowledge, no other study has used algometry when evaluating pressure pain over the masseter muscle in a gender perspective. Our finding that the gender of the examiner predicted PTol is in contrast with the only other study found using algometry in evaluation of pressure pain, although over the index and middle fingers, reporting that both men and women had higher levels of PTol, when the examiner was a woman [26]. Building on the findings from the present study suggesting that the gender of examiner does affect pain reporting, gender perspectives may play an important role also in the clinical settings. In contrast to healthy volunteers, care-seeking patients suffer from pain complaints that involve expectations on care provision and proper interaction with the clinician [27]. Previous studies have identified pain medicine as a field in health care affected by gender bias [28], with conclusive evidence showing that women are treated differently than men [29]. Women more often tend to be regarded as hysterical, emotional, and complaining [10] with chronic pain complaints regarded as psychological rather than somatic. However, for men with chronic pain, it may be the other way around, which can result in anxiety and depression being neglected [10]. Future studies in clinical settings and among patient samples are needed to evaluate the impact of the gender of the examiner on pain diagnostics and management.

### Strengths and limitations

In our study, we used the well-established and recommended technique of electric pressure algometry in the orofacial region for the evaluation of pain perception [30].

The pressure pain measurements in each participant were made by the same two examiners in a single visit. To reduce the effect of an increase in PPT after the first measurement [14, 31, 32], the order of the male and female examiner was randomized for each participant, as well as the order in which the participants were examined. To ensure reliability, examinations were performed in accordance with established guidelines regarding training and calibration of examiners [14]. However, in contrast to established guidelines, we did not control for female participants’ current phase in the menstrual cycle as was previously recommended [14]. In addition, confounding factors like smoking, diet, and physical activity were not addressed [33, 34]. Study samples based on voluntary participation could also potentially influence the results. However, since our data are based on repeated measures on the same day, we do not expect the factors described above to affect our main findings. With regard to pain reporting, cultural background is another additional factor that may influence the results. However, in relation to our aim, we did not include analysis of such subgroups and future studies will be needed to address these issues.

Participants examined by an examiner with high professional authority have shown higher pain tolerance to cold compared with student experimenters [35], which might have influenced our results as the examinations were carried out by dental students and not by experienced specialists in the field. In an attempt to minimize the effect of other influences aside from gender, the dental students in our study attended the same university, were of comparable age, wore clinical clothing, had a similar amount of clinical experience, and had the same amount of practice with the relevant research equipment. Another possible source of bias was that the study was not double-blinded since the examiners were closely intertwined in the experimental procedures. The participants were, however, not familiar with the aim of the study. Collectively, we believe that the present findings are representative for similar settings.

## Conclusion

The gender of the examiner influences pain reporting and perception in an experimental setting. This effect on pain perception related to gender of the examiner is probably related to normative gender behaviors rather than to biological alterations within the examined individual. The gender of the examiner may therefore also affect pain perception in clinical settings with potential implications for diagnostics.
